# Neuromuscular adaptations and sensorimotor integration following a unilateral transfemoral amputation

**DOI:** 10.1186/s12984-019-0586-9

**Published:** 2019-09-14

**Authors:** Claudia Ramos Claret, Georg W. Herget, Lukas Kouba, Daniel Wiest, Jochen Adler, Vinzenz von Tscharner, Thomas Stieglitz, Cristian Pasluosta

**Affiliations:** 1 Laboratory for Biomedical Microtechnology, Department of Microsystems Engineering, University of Freiburg, Freiburg, Germany; 2 Department of Orthopedics and Trauma Surgery, Medical Center, Faculty of Medicine, University of Freiburg, Freiburg, Germany; 3 Sanitätshaus Pfänder, Freiburg, Germany; 4 Human Performance Laboratory, University of Calgary, Calgary, Canada; 5 Bernstein Center Freiburg, University of Freiburg, Freiburg, Germany; 6 BrainLinks-BrainTools, University of Freiburg, Freiburg, Germany

**Keywords:** Postural control, Amputees, Sensory feedback, Prosthesis, Center of pressure

## Abstract

**Background:**

Following an amputation, the human postural control system develops neuromuscular adaptations to regain an effective postural control. We investigated the compensatory mechanisms behind these adaptations and how sensorimotor integration is affected after a lower-limb transfemoral amputation.

**Methods:**

Center of pressure (CoP) data of 12 unilateral transfemoral amputees and 12 age-matched able-bodied subjects were recorded during quiet standing with eyes open (EO) and closed (EC). CoP adjustments under each leg were recorded to study their contribution to posture control. The spatial structure of the CoP displacements was characterized by measuring the mean distance, the mean velocity of the CoP adjustments, and the sway area. The Entropic Half-Life (EnHL) quantifies the temporal structure of the CoP adjustments and was used to infer disrupted sensory feedback loops in amputees. We expanded the analysis with measures of weight-bearing imbalance and asymmetry, and with two standardized balance assessments, the Berg Balance Scale (BBS) and Timed Up-and-Go (TUG).

**Results:**

There was no difference in the EnHL values of amputees and controls when combining the contributions of both limbs (*p* = 0.754). However, amputees presented significant differences between the EnHL values of the intact and prosthetic limb (*p* <  0.001). Suppressing vision reduced the EnHL values of the intact (*p* = 0.001) and both legs (*p* = 0.028), but not in controls. Vision feedback in amputees also had a significant effect (increase) on the mean CoP distance (*p* <  0.001), CoP velocity (*p* <  0.001) and sway area (*p* = 0.007). Amputees presented an asymmetrical stance. The EnHL values of the intact limb in amputees were positively correlated to the BBS scores (EO: ρ = 0.43, EC: ρ = 0.44) and negatively correlated to the TUG times (EO: ρ = − 0.59, EC: ρ = − 0.69).

**Conclusion:**

These results suggest that besides the asymmetry in load distribution, there exist neuromuscular adaptations after an amputation, possibly related to the loss of sensory feedback and an altered sensorimotor integration. The EnHL values suggest that the somatosensory system predominates in the control of the intact leg. Further, suppressing the visual system caused instability in amputees, but had a minimal impact on the CoP dynamics of controls. These findings points toward the importance of providing somatosensory feedback in lower-limb prosthesis to reestablish a normal postural control.

**Trial registration:**

DRKS00015254, registered on September 20th, 2018.

## Introduction

The ability to maintain postural stability is one of the most important aspects of our daily life [1–3]. During upright standing, the human postural control system combines inputs from the visual, vestibular and somatosensory systems (i.e., proprioceptive and cutaneous sensations) to maintain the center of mass (CoM) over the base of support by adjusting the center of pressure (CoP).

Following a lower-limb amputation the ability to maintain balance is severely impaired. Since the stump cannot fully substitute the foot as a proprioceptive organ, the postural control system is reprogrammed and develops compensatory mechanisms to counteract weight-bearing asymmetries, loss of somatosensation, reduced size of support, and an increased joint stiffness [4–7]. These changes are reflected in alterations of neuromuscular motor outputs such as postural sway during upright standing [5]. While prosthetic devices are an alternative to partially circumvent this disability, commercially available technologies do not provide sensory feedback. The underlying adapted neuro-biomechanical mechanisms following a lower-limb amputation remain unclear.

Measurements of CoP adjustments over time during quiet standing is commonly used to evaluate the integrity of the postural control system [8–10]. CoP data are structured two dimensional outputs that carry information about the mechanisms involved in the control of bipedal unperturbed stance. The analysis of postural sway has been used in many studies to understand impairments of motor control in patients with pathologies that affect balance such as Parkinson’s disease [11, 12], Huntington’s disease [13], stroke [14, 15], and lower-limb amputees [4, 16–24]. Fluctuations in CoP time series are highly irregular and non-stationary [1, 3, 25], appearing as random variability but emerging from actual deterministic processes [15]. This complex variability observed in CoP time series is the result of the postural control system choosing one particular CoP adjustment from many possible options to maintain balance. This solution space is affected when sensory information is not available. Thus, using a measurement that is sensitive to the complex temporal relationship between CoP adjustments is important to study neuromuscular adaptations to postural challenges. Such changes of neuromuscular adaptations are likely to occur after losing a lower limb, where sensory feedback is disrupted.

Nonlinear methods such as approximate entropy (ApEn), sample entropy (SampEn) and fuzzy entropy (FuzzyEn) have been employed to quantify the regularity of the fluctuation in CoP time series [8, 26, 27]. Multiscale entropy (MSE) [28, 29], detrended fluctuation analysis (DFA) [30–32], and the Entropic Half-Life (EnHL) [33] were then developed to account for the multiple time scales inherent in physiologic processes. Applied to CoP data, the EnHL quantifies the time elapsed before previous CoP adjustments are no longer utilized by the postural control system to adjust the current CoP position. In contrast to the other multi-scale methods, the obtained EnHL value is in units of time. Changes in the EnHL of CoP adjustments reflects postural impairments resulting from a disrupted motor control, and captures immediate neuromuscular adaptations after training [11, 12, 26, 33–35].

The timing measured by the EnHL is the result of several feedback control loops acting to maintain balance and includes the delays from sensory pathways, the time required to process sensorimotor inputs, delays from the motor pathways, and delays from the musculoskeletal system. In the particular case of unilateral lower-limb amputees, one can assume that the delays from the motor pathways, sensorimotor processing and the musculoskeletal system of the unaffected limb remains unaltered after the amputation. Thus, by comparing the time scale provided by the EnHL for amputees and able-bodied subjects, it is possible to estimate on which sensory modality the postural control system preferably relies on.

After an unilateral transfemoral amputation, compensatory mechanisms were observed in the intact limb and seem to be essential to maintain a stable balance during quiet upright standing [20, 24]. Curtze and colleagues analyzed the contribution of the prosthetic and intact leg to balance control during upright stance after an external perturbation [36]. They recorded the postural sway with two force plates to obtain CoP signals from each leg independently. Their study revealed that the intact leg compensates for the missing ankle in the anterior-posterior (AP) direction by increasing the ankle movement. In the medio-lateral (ML) direction, amputees experienced less limitations since they used a hip strategy (as also observed in healthy controls) to recover from perturbations [35]. Hlavackova and colleagues observed that the body weight of unilateral lower-limb amputees during unperturbed standing shifted towards the intact leg, which resulted in a larger CoP velocity and sway area compared to the prosthetic leg [5]. Additionally, the SampEn of the CoP adjustments under the intact leg was lower than under the prosthetic limb, reflecting a more regular CoP signal.

Compensatory activity by the intact limb were also observed in patients with unilateral below-knee amputations [23, 24]. Using two force plates, Isakov and Mizrahi measured the ground reaction forces, showing that in this population also most of the standing control is performed by the intact limb with AP as the main direction of control [23]. Measures of mean sway position, root mean squared (RMS) amplitude of the CoP adjustments and RMS of the CoP velocity were obtained from patients with below-knee (*n* = 3), through-knee (*n* = 3) and above-knee (*n* = 3) amputations before and after rehabilitation [24]. Less dependency on visual information was observed in the group of amputees after rehabilitation, with CoP measures approaching normal values. This suggests a reorganization of postural control after the amputation and an integration of sensory information from the amputated limb after rehabilitation.

The aim of this study was to investigate the neuromuscular adaptations resulting from a disrupted sensorimotor system that was caused by a unilateral lower-limb amputation. We exploited the fact that the EnHL measures short-term correlations in CoP data in units of time. This allowed to assess the dynamics of the CoP adjustments after an amputation, which reflects the altered timing of the different sensory systems involved in balance control. We expected to observe the short-term correlations of the CoP adjustments associated with the different time delays observed in visual and proprioception feedback control loops [37]. As in previous studies with unilateral amputees, a greater contribution to balance of the intact leg was expected to circumvent the loss of somatosensory feedback as well as a strong reliance on the visual system. We complimented the analysis with measures of spatial properties of the CoP signals, assessments of load distribution asymmetry, and outcomes from standardized tests that are used to evaluate balance control in clinical settings.

## Methods

### Participants

A total of 12 unilateral lower-limb amputees (age: 46.08 ± 13.8 yr.; height: 173.67 ± 11.84 cm; weight: 71.17 ± 14.04 kg) and 12 age-matched able bodied control participants (age: 40.67 ± 12.44 yr.; height: 175.92 ± 9.38 cm; weight: 74.67 ± 10.32 kg) were recruited by Pfänder Orthopedics, Freiburg, Germany. All amputee patients presented unilateral transfemoral amputations. Subjects with neurological, cardiovascular diseases, and other orthopedic conditions were excluded from the study. All participants had normal or corrected-to-normal vision. The characteristics of the participating amputees and healthy controls are shown in Table 1. Limb dominance was defined as the leg that is mainly used for propulsion (forward acceleration of the body’s center of gravity). We verbally asked the participants: “Which leg are you going off first? For climbing stairs, which leg you move first or with which leg you step on the first step”. The dominant leg was defined as the leading leg, i.e. first leg to step forward, in both of these scenarios. In this study, the type of prostheses, causes of amputation, and stump lengths differed among the participants with transfemoral amputations. The Genium and C-leg models (Ottobock, Duderstadt, Germany) have an electronically-controlled “stance phase mode”, which increase the flexion resistance of the knee joint. As soon as the pressure distribution changes, e.g., while a step forward is made with the healthy leg, sensors in the prosthetic leg recognize the changed pressure distribution and release the joint. The Synergy model (Neuhoff, Nürnberg, Germany) also has a stance phase protection, which is performed via hydraulic control. The length of the prosthetic limb did not differ more than 5 mm from the length of the intact limb, measured by the orthotists/prosthetists, with a minimal shorter prosthetic limb to improve the swing phase. The size of the prosthetic foot is adapted such that it fits securely into the patient’s shoe. Participants had a dynamic foot (e.g., the Triton model from Ottobock or alike). Participants were shod.

**Table 1:** Demographics of the participants

Subject	Age in years	Gender	Weight in kg	Height in cm	Amputated / non-dominant leg	Years since amputation	Prosthesis Type	Length of stump in cm	Etiology
1	55	M	72	183	Right	36	O Genium	22	Tumor
2	62	M	65	187	Left	34	O Genium	28	Trauma
3	67	M	69	170	Left	50	O C-leg	21	Tumor
4	39	M	70	188	Left	28	O Genium	24	Tumor
5	49	F	65	158	Right	33	O C-leg	25	Tumor
6	27	F	64	165	Left	22	O Genium	13	Trauma
7	56	F	61	163	Left	50	O C-leg	23	Tumor
8	26	F	48	168	Right	1	NH Synergy	14	Sepsis
9	37	M	100	185	Right	20	O Genium	28	Trauma
10	32	M	97	186	Right	4	O Genium	25	Trauma
11	54	M	81	178	Left	36	O Genium	24	Trauma
12	49	F	54	153	Left	28	O Genium	25	Tumor
13	31	F	83	183	Left	–	–	–	–
14	40	M	80	179	Left	–	–	–	–
15	22	F	58	169	Left	–	–	–	–
16	34	F	60	175	Left	–	–	–	–
17	56	F	70	175	Left	–	–	–	–
18	39	F	85	164	Left	–	–	–	–
19	62	M	87	179	Left	–	–	–	–
20	37	M	75	160	Left	–	–	–	–
21	54	F	73	175	Right	–	–	–	–
22	28	M	73	190	Left	–	–	–	–
23	30	M	90	193	Left	–	–	–	–
24	55	F	62	169	Left	–	–	–	–

*M* Male, *F* Female, *O* Ottobock, *NH* Neuhoff

### Experimental design

Participants were subjected to static posturographic tests and two standardized balance assessments, the Berg Balance Scale (BBS) and Timed Up-and-Go (TUG) tests [38, 39]. For the static posturographic test, CoP data were recorded at 60 Hz using a FDM-S force platform (zebris Medical GmbH) during six 30-s trials of quiet standing, three with eyes open (EO) and three with eyes closed (EC). Participants were instructed to stand quietly with their feet separated to match shoulder to shoulder distance and their arms hanging loosely close to the body. A mark was placed on the force plate, which indicates the place where the middle point between the feet should be located. Orientation of the feet to each other was left at their discretion. During the EO condition, participants were instructed to look at a cross mark placed at their eye level and at a distance of 1.5 m. The same instruction was issued to all participants to avoid discrepancies in the CoP dynamics [40]. The trials were randomized with short rest breaks in between. The BBS consists of 14 common, everyday tasks that the subjects performed in order to evaluate their static and dynamic balance abilities. According to the degree of success, each task is graded with a score from 0 to 4, 0 meaning the subject was unable to complete the task and 4 that he or she completed the task successfully. The outcome of the test is the sum across all the scores [39, 41, 42]. The TUG test evaluates the dynamic stability of the participants by recording the time they need to rise from a chair, walk three meters, turn around (180 degrees), walk the three meters back and sit down [42, 43].

### Data analysis

The CoP data were filtered with a wavelet bandpass filter. The cut-off frequencies of 0.15 and 10 Hz were used to remove low frequency drift and high frequency noise [11, 12, 33–35]. In addition, phase-randomized surrogates of the CoP data were computed using the Amplitude-Adjusted Fourier Transform (AAFT) method to reject the possibility that the CoP signal comes from a random process [8, 11, 12]. The spatial structure was characterized by the mean distance from the CoP mean ($\overline{\mathrm{DIST}}$), the mean velocity ($\overline{\mathrm{VEL}}$) and the 95% confidence ellipse area (AREA_CE_), which were calculated according to the formulas published by Prieto et al. [9]. To further characterize the postural control system, the weight bearing imbalance factor (WBI) and the stance angle (θ) were calculated. The WBI quantifies the force difference between each foot [2, 23]. In this study, θ was defined as the angle between the foot’s y-axis (AP direction) and the global y-axis. We also analyzed the distribution of body weight over each of the participants’ legs, computed as the average force distribution on each foot, as well as the force distribution on each fore- and rear-foot. Asymmetrical body weight distributions either between legs or between fore and rear foot areas were herein documented as shifts in load distribution.

The EnHL was then calculated on the filtered CoP and the filtered surrogate data following the methodology described in previous work [11, 12, 26, 33–35, 44, 45]. In short, the times series was first gradually randomized up to 25 rescales (corresponding to timescales between 10 and 250 ms) using the reshape scale method [33, 44]. The FuzzyEn (m = 3, r = 0.7, expo = 5, see Additional file 1) was then calculated for each randomization step and normalized with respect to its maximum value. The FuzzyEn was chosen for its robustness and to overcome the limitation of the Heaviside step function used to calculate the similarity degree between vectors in the approximate and sample entropy [46–48]. Further, with FuzzyEn the effect of counting noise can be alleviated [26]. The EnHL corresponded to the time scale at which the normalized FuzzyEn reaches half of its maximal value (see Additional file 1 for more details). All the CoP measures where averaged across the 3 trials for each visual condition. Data analysis was performed in Matlab version 2017a (MathWorks, Inc., MA, United States).

### Statistical analysis

A four-way mixed ANOVA was implemented for the $\overline{\mathrm{DIST}}$, $\overline{\mathrm{VEL}}$, AREA_CE_, WBI, and EnHL values to test for between-subject factors of *Group* (two levels, amputee and control), *Direction* (two levels, ML and AP) and *Leg* (three levels, intact/dominant, amputated/non-dominant and both), and the within-subject factor *Condition* (two levels, EO and EC). In case of significant effect or interaction, a post hoc test with Bonferroni correction was conducted. The significance level was set to α = 0.05. All statistical analysis in this study were performed using R 3.5.0 (R Core Team, New Zealand).

## Results

### Clinical assessments

On this small cohort of patients, amputees with the Ottobock model Genium tended to score better in the clinical assessments than the amputees with the Ottobock C-Leg (t-test, BBS: *p* = 0.001, TUG: *p* = 0.0019) and presented a more symmetrical load distribution (*p* <  0.001). This comparison should be confirmed in a larger cohort of patients. Patients with a traumatic amputation completed the TUG test with significantly less time (*p* <  0.001) and presented a significantly lower WBI (*p* <  0.001) than patients with non-traumatic amputations. There were no significant correlations between the stump length and any of the measurements. The BBS score was positively (ρ = 0.442, *p* <  0.001) and the TUG time negatively (ρ = 0.-0.361, *p* <  0.001) correlated with the time since the amputation.

### Posturography assessments

**Fig. 1:**
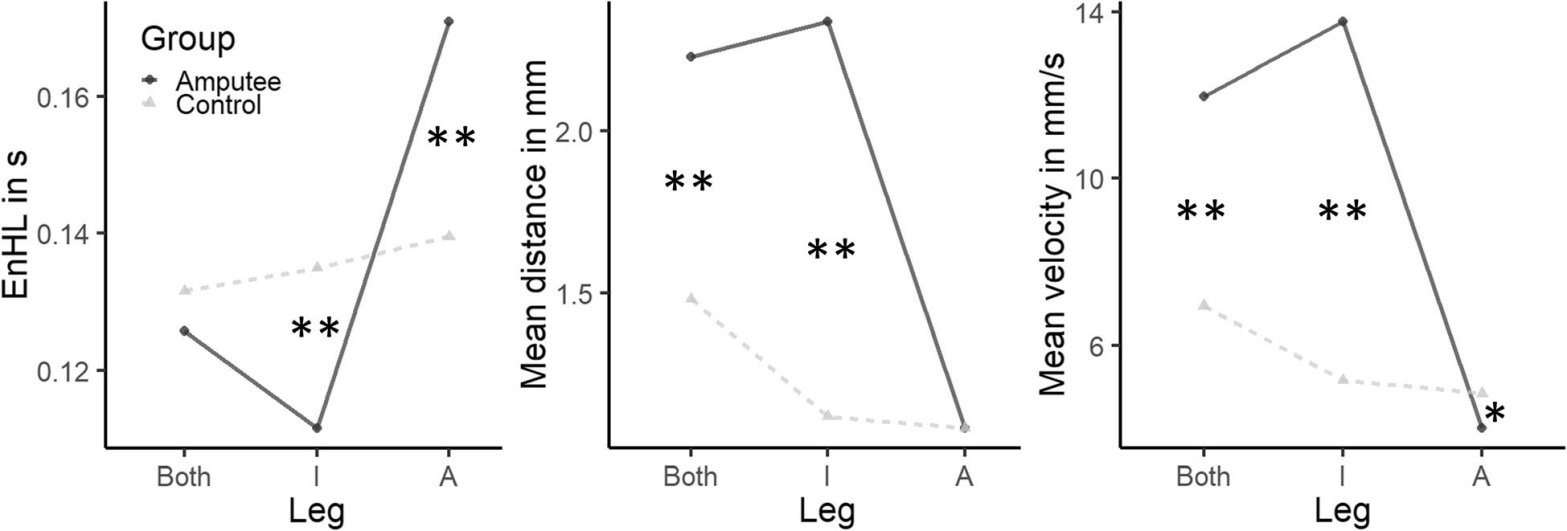
*Group* x *Leg* interaction of the EnHL values (left), $\overline{\mathrm{DIST}}$ (middle) and $\overline{\mathrm{VEL}}$ (right). The significant differences between groups are marked with an asterisk (*: *p* < 0.05, **: *p* < 0.001). (I: intact leg, in case of controls dominant leg, A: amputated leg, in case of controls non-dominant leg)

The EnHL values of the original CoP data were significantly larger (F = 411.54, *p* <  0.001) than the ones from the surrogate CoP data, confirming the non-random nature of the CoP time series. There was no significant *Group* effect (F = 0.021, *p* = 0.884, Table 2) on the EnHL values but the structure of the CoP pattern did differ significantly between amputees and controls ($\overline{\mathrm{DIST}}$: *p* <  0.001, $\overline{\mathrm{VEL}}$: *p* <  0.001, AREA_CE_: *p* = 0.007, Table 2). A significant *Leg* effect was observed (F = 17.383, *p* <  0.001), and a significant *Group* x *Leg* interaction was also present (F = 11.574, *p* <  0.001). While there was no significant difference between the limbs in the group of controls, the EnHL values of the CoP data produced by the prosthetic leg were significantly larger than the EnHL values of the intact leg (prosthetic: 171 ± 29 ms, intact: 111 ± 27 ms, *p* <  0.001). Further, the intact leg of the group of amputees presented significantly lower EnHL values than the dominant and non-dominant leg of controls (dominant: 135 ± 31 ms, non-dominant: 140 ± 39 ms, *p* <  001). This interaction was further analyzed using post hoc tests with Bonferroni correction (Fig. 1 – left). This significant interaction was also present in the $\overline{\mathrm{DIST}}$ (F = 7.59, *p* = 0.001, Fig. 1 - left) and $\overline{\mathrm{VEL}}$ (F = 7.854, p = 0.001, Fig. 1 - middle). The post hoc tests revealed no significant differences in the $\overline{\mathrm{DIST}}$ of any limb of the group of controls and the prosthetic limb of amputees (*p* = 0.99). However, the $\overline{\mathrm{DIST}}$ of the intact leg of amputees was significantly larger than the $\overline{\mathrm{DIST}}$ of the dominant and non-dominant leg of controls (*p* <  0.001). The $\overline{\mathrm{VEL}}$ presented a similar trend than the $\overline{\mathrm{DIST}}$, with an additional significant difference between non-dominant and prosthetic leg (*p* <  0.05, Fig. 1 - right).

**Table 2:** Results of the four-way mixed ANOVA performed on the EnHL values of the original CoP data. Statistical significant are marked in boldface (α = 0.05)

Effect or interaction	EnHL		$\overline{\mathrm{DIST}}$		$\overline{\mathrm{VEL}}$		AREA_CE_	
	*F*-value	*p*-value	*F*-value	*p*-value	*F*-value	*p*-value	*F*-value	*p*-value
Group	0.021	0.884	**25.847**	**< 0.001**	**18.868**	**< 0.001**	**7.808**	**0.007**
Direction	3.240	0.074	**13.750**	**< 0.001**	**11.685**	**< 0.001**	3.698	0.075
Leg	**17.383**	**< 0.001**	**154.577**	**< 0.001**	**56.189**	**< 0.001**	NA	NA
Condition	3.240	0.074	**127.404**	**< 0.001**	**91.044**	**< 0.001**	**11.249**	**0.001**
Group x Direction	2.568	0.111	**7.590**	**0.001**	**7.854**	**0.001**	2.579	0.083
Group x Leg	**11.574**	**< 0.001**	2.949	0.088	**4.303**	**0.040**	NA	NA
Direction x Leg	0.070	0.932	**7.556**	**0.001**	**5.833**	**0.004**	NA	NA
Group x Condition	**4.746**	**0.031**	**30.975**	**< 0.001**	**33.767**	**< 0.001**	**8.613**	**0.005**
Direction x Condition	0.282	0.596	**6.825**	**0.002**	**10.465**	**< 0.001**	2.735	0.072
Leg x Condition	2.199	0.115	**47.486**	**< 0.001**	**32.890**	**< 0.001**	NA	NA
Group x Direction x Leg	0.191	0.826	**4.243**	**0.016**	**3.934**	**0.022**	NA	NA
Group x Direction x Condition	1.004	0.318	**6.047**	**0.003**	**9.176**	**< 0.001**	2.675	0.076
Group x Leg x Condition	1.191	0.307	**9.519**	**0.002**	**11.898**	**0.001**	NA	NA
Direction x Leg x Condition	0.348	0.707	2.421	0.093	**3.854**	**0.024**	NA	NA
Group x Direction x Leg x Condition	0.234	0.791	2.655	0.074	**3.733**	**0.026**	NA	NA

No significant differences in the EnHL values of the CoP time series in the ML and AP directions were observed (F = 3.240, *p* = 0.074, Table 2). While EnHL values did not differ significantly between the EO and EC conditions (F = 3.240, p = 0.074), a significant *Group* x *Condition* interaction was observed (F = 4.746, *p* = 0.031, Fig. 3). The EnHL values during EC were significantly lower than the EnHL values in the EO condition within the group of amputees (*p* = 0.003, Fig. 3 - left). However, no significant changes were observed in the EnHL values of control after removing visual feedback (*p* = 0.800, Fig. 3 - left). The spatial measures increased significantly for both groups during the EC condition (Table 2). The WBI also increased significantly with EC in both groups (Amputees: *p* <  0.001, Controls: *p* = 0.002). In the group of controls, there were no significant differences without vision between the EnHL values of the dominant (I, *p* = 0.690, Fig. 3 - right), non-dominant (A, *p* = 0.670, Fig. 3 - right) and both legs (Both, *p* = 0.640, Fig. 3 - right). However, there were significant differences in the group of amputees. While the EnHL values of the prosthetic leg (A, *p* = 0.720, Fig. 3 - right) did not change significantly during the EC condition, the EnHL values of the intact and both legs decreased when the visual information was suppressed (I: *p* = 0.001, Both: *p* = 0.028, Fig. 3 - right). The EnHL values during the EO condition were 134 ± 32 ms for amputees and 133 ± 36 ms for controls. During the EC condition, amputees presented an EnHL value of 118 ± 25 ms and controls of 130 ± 34 ms.

While the stance angle of the dominant and non-dominant leg of controls did not differ significantly (*p* = 0.09), the stance angle of the intact leg was significantly larger than that of the prosthetic leg in the group of amputees (*p* = 0.002). This resulted in a stance angle of both feet of θ = 12.1 ± 7.9° in the amputees and of θ = 3.3 ± 2.3° in controls. In the group of amputees, the combined stance angle depended mostly on the stance angle of the intact leg. Amputees had an asymmetrical load distribution with most of their weight shifted towards their intact leg (with EO 56% of the average force was distributed toward the intact leg and with EC 61%). In contrast, controls had a symmetrical load distribution, which did not change significantly between EO and EC (approximately 50%). There was a significant difference between the WBI of the two groups (F = 39.330, *p* <  0.001). Further, the load under the prosthetic limb was shifted toward the forefoot in amputees (with EO 69% and with EC 65%). In contrast, with controls whom shifted their weight toward the rear foot (with EO and EC approximately 58.5%). The WBI factor was positively correlated (EO: ρ = 0.357, p = 0.002, EC: ρ = 0.296, *p* = 0.012, Fig. 4) in amputees and negatively correlated in controls (EO: ρ = − 0.284, *p* = 0.016, EC: ρ = − 0.454, *p* <  0.001, Fig. 4) with the TUG time.

## Discussion

The aim of this study was to evaluate the neuromuscular adaptations and control strategies of unilateral transfemoral amputees to maintain balance during quiet standing. We estimated the time scale at which the postural control system of amputees acts during quiet standing in comparison to able-bodied subjects. This provides insights on which sensory feedback amputees rely more after losing somatosensation from the amputated leg. Specifically, we investigated the reliance on visual versus proprioception information in the control of the intact limb. The decreased EnHL values observed in the intact leg of amputees compared to able-body controls points to a predominant usage of proprioception inputs as the time scale at which the postural control acts was shorter. Yet, the time scale at which the postural control acted in unilateral transfemoral amputees wearing a prosthesis was similar to able-bodied subjects when looking at the contribution of both legs to the CoP dynamics. This suggests that the intact limb of amputees compensates for the impaired postural control of the prosthetic leg.

**Fig. 2:**
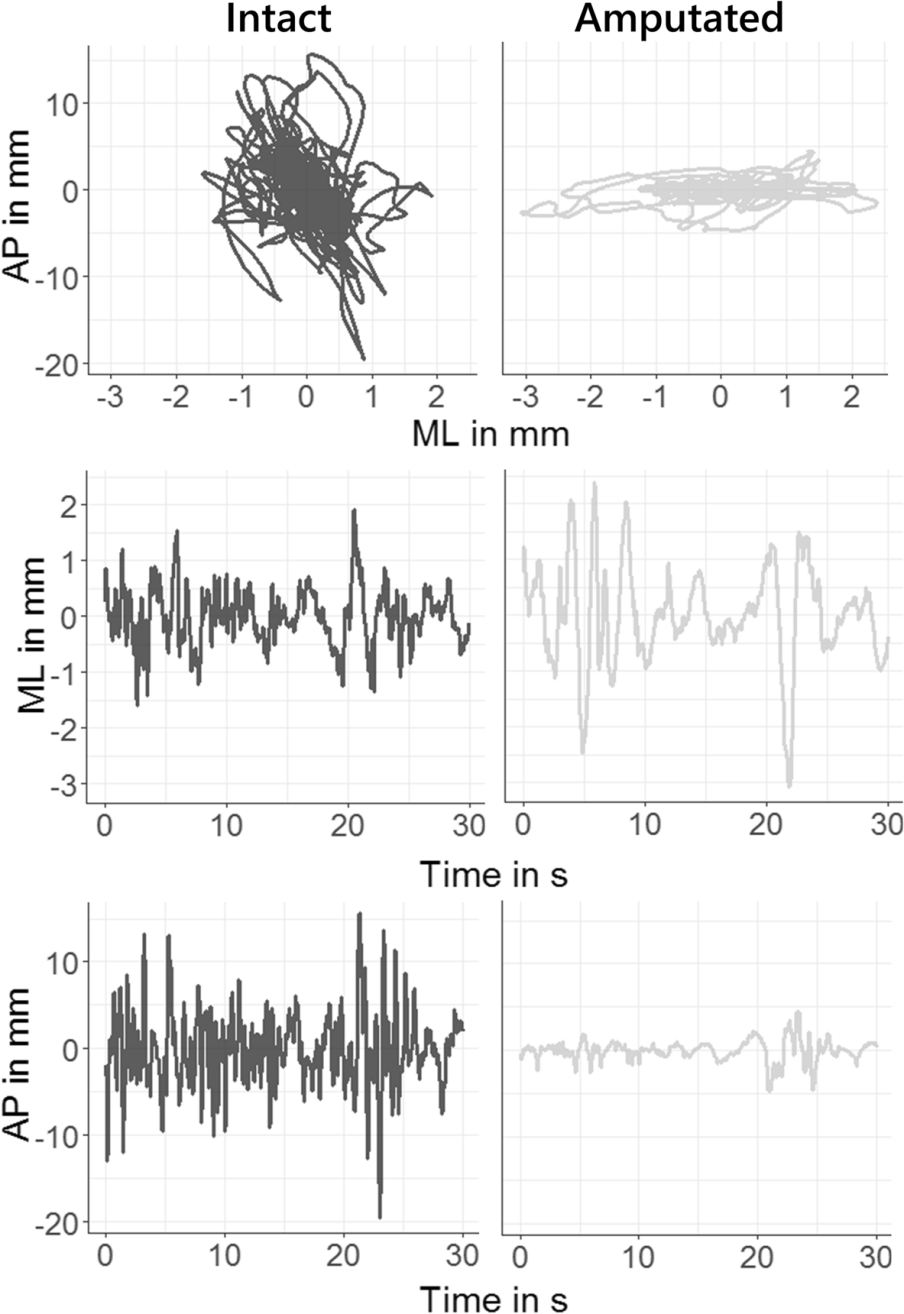
CoP path of the intact (left) and amputated (right) leg of an amputee in the AP and ML direction

Even though the prosthetic leg serves to support the weight during upright standing, the intact leg plays a prominent role in the control of balance. This has been reported consistently in the literature [5, 20, 23]. Yet, the active compensatory role of the intact limb is not sufficient to maintain balance as efficient as in the group of controls, which has been observed in previous work [2, 17, 22]. Our results suggest some further possible explanations to this control deficiency. The CoP adjustments of the intact leg seems to be controlled within shorter time-scales (evidenced by lower EnHL values) and less stringently (producing larger $\overline{\mathrm{DIST}}$ and $\overline{\mathrm{VEL}}$) than its prosthetic counterpart (Fig. 2). Further, the relatively larger mechanical stiffness of the prosthetic limb (evidenced by smaller $\overline{\mathrm{DIST}}$ and $\overline{\mathrm{VEL}}$ in the prosthetic leg) may limit movements in the dorsiflexion and plantarflexion directions, as it has been previously reported [1, 17, 49].

**Fig. 3:**
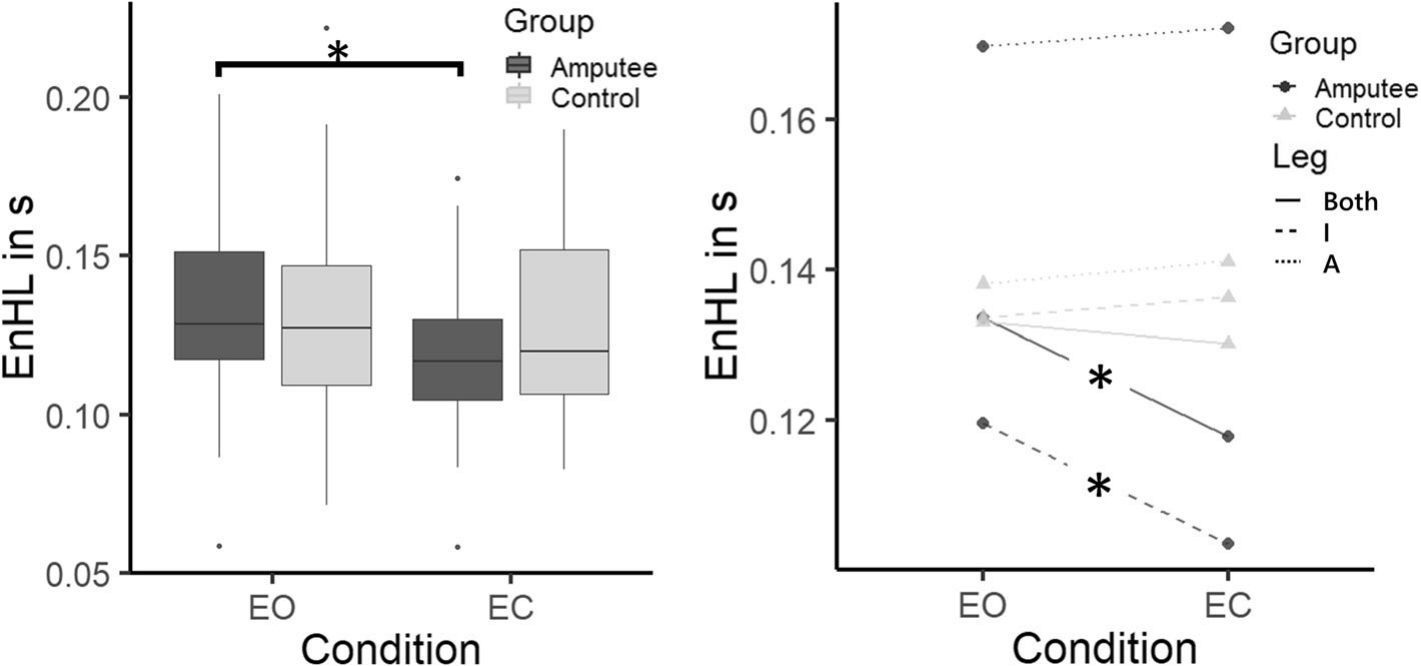
Box plot of the *Condition* x *Group* interaction of the EnHL values considering the contributions of both legs (left), and interaction plot of the EnHL values of each limb independently and each group during EO and EC condition. Box plots describe the median (line inside the box) and the 1st and 3rd quartiles (box hinges). The box whiskers represent the largest (or smallest) data value but no larger (or smaller) than 1.5 times the inter quartile range. Values larger (or smaller) than the whiskers are represented by dots. The significant differences are marked with an asterisk (*: *p* < 0.05). (I: intact leg, in case of controls dominant leg, A: amputated leg, in case of controls non-dominant leg)

Delays associated with vision follows proprioception delays by approximately 40 to 50 ms [37]. The EnHL values provide information about the response time of the postural control system to past CoP adjustments. Assuming that the delays in the descending (motor) pathways to the intact leg, the delays associated with sensorimotor processing and the musculoskeletal systems remain unaltered after an unilateral leg amputation, the lower EnHL value observed in the intact leg of amputees (25 to 30 ms shorter than any leg of the control subjects, Fig. 2 – left) points to a larger involvement of the proprioceptive system, which become more evident when vision was removed (Fig. 3 – right).

With eyes closed, the adjustments of the CoP in amputees were faster with increased CoP excursion, failing to maintain a quiet balance as instructed. During the EC condition the postural system may have been able to perform more CoP adjustments in less time (i.e., with less delay), and thus increasing the sway velocity. The increase of postural sway under the EC condition with respect to EO has also been reported in other studies [8–10], and some studies highlighted the increase of postural sway in amputees in contrast to able-bodied subjects [17, 21]. In our study, the EnHL value of the prosthetic leg did not change significantly when vision was suppressed (Fig. 3 – right). However, the suppression of the visual system did affect the control of the intact leg, which was reflected in a decrease in the EnHL values during the EC condition, suggesting a further involvement of the proprioceptive system. Additionally, the EnHL value of both legs combined was shifted toward the values observed in the intact leg, showing a minor impact of the prosthetic limb on the resulting CoP dynamics. Taking these results together, it seems that the postural control system in the group of amputees further increased its reliance on the proprioceptive system when visual input was compromised, especially in the control of the intact leg. Geurts and colleagues observed less dependency on visual feedback to control posture after rehabilitation training [24], which points to the importance of training with an emphasis on the use of somatosensory information to improve balance control in amputees.

**Fig. 4:**
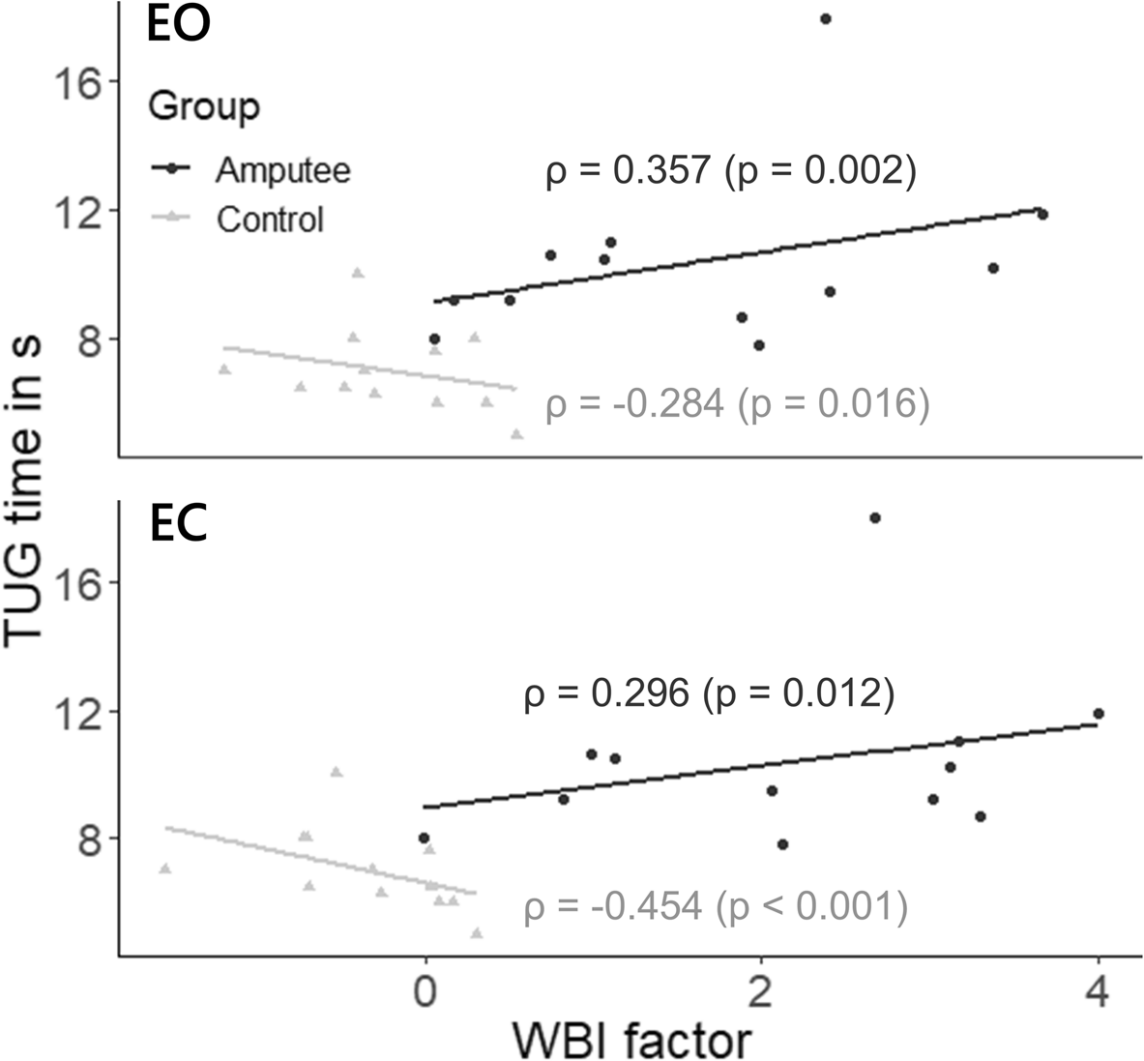
Scatter plot of the WBI values and the TUG times. The correlation between the TUG times and WBI factor is positive in amputees and negative in controls. The lines represent the regression lines. The corresponding Pearson coefficient ρ and *p*-values are presented in each plot

We did not observe correlations between the CoP dynamics of amputees and the number of years since the amputation. This was also the case for the mean CoP distance, mean CoP velocity and CoP area. This may indicate that the observed neuromuscular adaptations are due to the mechanics of using a prosthetic leg rather than a cortical remapping. However, to confirm these results a within-subject monitoring in longitudinal studies of these postural control measures over time is required. Amputees did improve their overall control of balance (higher BBS scores and reduced TUG times) with increasing time since the amputation. Even though this again should be confirmed with a within-subject follow-up, these results further indicate that amputees develop compensatory mechanisms (as discussed before, mostly in their intact leg) to improve their balance control in such a degree that they can complete the tasks almost completely independently. However, as also shown in previous studies [7, 20–22, 49], amputees presented a large weight-bearing imbalance and an asymmetric stance. This asymmetry may associate with long-term neuromuscular problems such as back and limb pain, vascular diseases and premature arthritis [21, 22]. The results also suggest that the WBI impacts the TUG performance of amputees (Fig. 4). This reflects the importance of the stance and load distribution symmetry for the completion of simple activities.

The current study only analyzed postural control during quiet standing. Future research should investigate balance control during perturbed stance to get more insights into the mechanisms involved in the observed neuromuscular adaptations. We did not control for the alignment of the prosthesis, which may have influenced the measures of the stance angle. However, patients had a stable knee joint during stance and did not have to perform an internal rotation of the hip for stabilization. The weight of the prosthetic limb is usually lower than a real limb, which may also play a role during balance control. Further, this study reported balance performance of transfemoral lower limb amputees with different prosthesis types, causes of amputation and stump lengths. Amputees with different etiology and prosthesis type presented significantly different CoP dynamics. However, in our study the stump length did not correlate with changes in the CoP adjustments. Therefore, a larger sample size with more homogenous clinical characteristics should be further investigated.

## Conclusion

Unilateral transfemoral amputees present an impaired postural control, which can be observed in the spatial and temporal structure of their CoP dynamics during quiet standing. The intact leg of amputees seems to compensate for the mechanical limitations and loss of somatosensation in the prosthetic limb. The EnHL allowed to further investigate the time scale at which the postural control system regulates the CoP adjustments during quiet standing, providing information about the sensory modalities that predominate during balance control after a lower-limb amputation. This work established an objective baseline to evaluate the next generation of prostheses with sensory feedback either via surface stimulation or via neural implants.

## Supplementary information

Additional file 1: Computation of the FuzzyEn, the EnHL and the reshape scale method. (PDF 452 kb)

## Data Availability

The datasets generated during and/or analysed during the current study are available from the corresponding authors on reasonable request.

## Abbreviations

AREA_CE_: 95% confidence ellipse area
$\overline{\mathrm{DIST}}$: Mean distance from the CoP mean
$\overline{\mathrm{VEL}}$: Mean CoP velocity
AAFT: Amplitude-Adjusted Fourier Transform
ANOVA: Analysis of variance
AP: Anterior-posterior
ApEn: Approximate entropy
BBS: Berg Balance Scale
CoM: Center of mass
CoP: Center of pressure
DFA: Detrended fluctuation analysis
EC: Eyes closed
EnHL: Entropic Half-life
EO: Eyes open
FuzzyEn: Fuzzy entropy
ML: Medio-lateral
MSE: Multiscale entropy
SampEn: Sample entropy
TUG: Timed Up-and-Go
WBI: Weight bearing imbalance factor
θ: Stance angle
