# Supplementary material for: Neuromuscular adaptations and sensorimotor integration following a unilateral transfemoral amputation
Source: J Neuroeng Rehabil. 2019 Sep 14;16:115. doi: 10.1186/s12984-019-0586-9 (PMC6744715; doi:10.1186/s12984-019-0586-9)
Supplement: Supplementary file 1 — Computation of the FuzzyEn, the EnHL and the reshape scale method. (PDF 452 kb) [file 12984_2019_586_MOESM1_ESM.pdf]

Neuromuscular adaptations and sensorimotor integration following a unilateral transfemoral amputation

Claudia Ramos Claret[1], Georg W Herget[2], Lukas Kouba[2], Daniel Wiest[3], Jochen Adler[3], Vinzenz von Tscharner[4], Thomas Stieglitz[1,5,6†], Cristian Pasluosta[1†]

1 Fuzzy Entropy

Fuzzy entropy (FuzzyEn) was computed following the methodology described by Chen and colleagues [1]. We describe briefly here the steps to compute the FuzzyEn of a time series $\{u(i), 1 \leq i \leq N\}$. First, given some factor m, q sub-sequences from $u(i)$ (with $q = 1, \dots, N - m + 1$) are created and detrended (Equation 1 and 2). Then, the similarity between these sequences are computed through a fuzzy function (Equation 3 and 4, in this work an exponential function was used as in Chen et al. [1]). The function Φ^{m} is then computed. This procedure is repeated but for $m + 1$ points. The FuzzyEn is then defined by Equation 6 and estimated for finite time series by Equation 7.

$$X_q^m = \{u(q), u(q+1), \dots, u(q+m-1)\} - u0(q) \tag{1}$$

$$u0(q) = \frac{1}{m} \sum_{k=0}^{m-k} u(q+k) \tag{2}$$

$$u\left(d_{ij}^m, r, expo\right) = \exp\left(\frac{-\left(d_{ij}^m\right)^{\mathrm{expo}}}{\mathrm{r}}\right) \tag{3}$$

$$d_{ij}^m = \max_{k \in (0, m-1)} |u(i+k) - u0(q) - u(j+k) - u0(q)| \tag{4}$$

$$\Phi^{\mathrm{m}}(r, expo) = \frac{1}{\mathrm{N\text{-}m}} \sum_{\mathrm{i=1}}^{\mathrm{N\text{-}m}} \left(\frac{1}{\mathrm{N\text{-}m\text{-}1}} \sum_{\substack{\mathrm{j=1,} \\ \mathrm{i \neq 1}}}^{\mathrm{N\text{-}m}} \mathrm{u}\left(d_{ij}^m, \mathrm{r}, \mathrm{expo}\right) \right) \tag{5}$$

$$\text{FuzzyEn(m, r, expo)} = \lim_{N\to\infty}\left(\ln \Phi^{m}(r, expo) - \ln \Phi^{m+1}(r, expo)\right) \tag{6}$$

$$\text{FuzzyEn(m, r, expo)} = \ln \Phi^{m}(r, expo) - \ln \Phi^{m+1}(r, expo) \tag{7}$$

2 The Entropic Half-Life

To calculate the Entropic Half-life (EnHL), we followed the methodology described by Zandiyeh and von Tscharner [2]. We describe the procedure as follows. The time series is first gradually randomized by reshaping and rearranging the signal at a specific time scale, τ, using the reshape scale method introduced in the next subsection. For each time scale, the entropy is calculated (in this work the FuzzyEn was used). The result are increasing values of entropy for each increment of τ. The resulted entropy values are normalized with respect to the its maximum (i.e., when the time series is fully randomized). The EnHL is then defined as the time scale at which the normalized FuzzyEn values reaches half of its maximal value (Figure S1). In other words, the EnHL is the time scale at which the reshaped time series transitions from a deterministic to an uncorrelated behaviour.

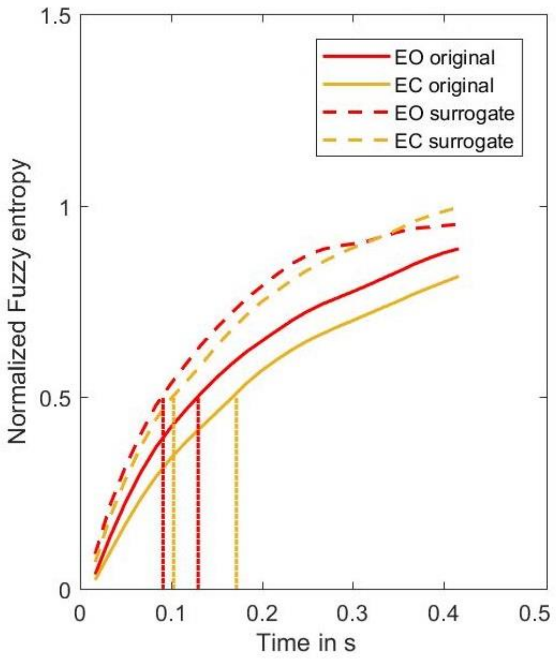

Figure S1: A representative example of how the EnHL is computed for the EO and EC condition and for the original and surrogate CoP data. The normalized fuzzy entropy is shown as a function of the reshape scale factor.

3 Reshape Scale Method

In the reshape scale method the data points of a time series are rearranged in a way that the time distance between consecutive points increases with increasing values of τ [2]. This results in an increasing randomness of the time series with increasing τ. Mathematically, the reshape scale method can be described with Equations (4) and (5), where from a time series $x(i), 1 \leq i \leq N$, the sequences Y_i forms the new reshaped time series TS_{new}.

$$Y_i = [x(i+0\times\tau), x(i+1\times\tau), x(i+2\times\tau), x(i+3\times\tau), \dots, x(i+N\times\tau)] \text{ with } i = 1, 2, \dots, \tau \quad (4)$$

$$TS_{new} = [Y_1, Y_2, \dots, Y_\tau] \quad (5)$$

With a rescale factor of $\tau = 1$, TS_{new} equals to the original time series.

References

[1] W. Chen, Z. Wang, H. Xie, and W. Yu, “Characterization of surface EMG signal based on fuzzy entropy.,” *IEEE Trans. Neural Syst. Rehabil. Eng.*, vol. 15, no. 2, pp. 266–272, 2007.

[2] P. Zandiyeh and V. Von Tscharner, “Reshape scale method: A novel multi scale entropic analysis approach,” *Phys. A Stat. Mech. its Appl.*, vol. 392, no. 24, pp. 6265–6272, 2013.
